# Efficient Elimination of Cancer Cells by Deoxyglucose-ABT-263/737 Combination Therapy

**DOI:** 10.1371/journal.pone.0024102

**Published:** 2011-09-19

**Authors:** Ryuji Yamaguchi, Edith Janssen, Guy Perkins, Mark Ellisman, Shinichi Kitada, John C. Reed

**Affiliations:** 1 Program of Cell Death and Apoptosis, Sanford-Burnham Medical Research Institute, La Jolla, California, United States of America; 2 Division of Molecular Immunology, Cincinnati Children's Hospital Medical Center, Cincinnati, Ohio, United States of America; 3 Department of Neurosciences, University of California San Diego, La Jolla, California, United States of America; 4 Department of Molecular Biology, Kyushu University Medical School, Fukuoka, Japan; University of Illinois at Chicago, United States of America

## Abstract

As single agents, ABT-263 and ABT-737 (ABT), molecular antagonists of the Bcl-2 family, bind tightly to Bcl-2, Bcl-xL and Bcl-w, but not to Mcl-1, and induce apoptosis only in limited cell types. The compound 2-deoxyglucose (2DG), in contrast, partially blocks glycolysis, slowing cell growth but rarely causing cell death. Injected into an animal, 2DG accumulates predominantly in tumors but does not harm other tissues. However, when cells that were highly resistant to ABT were pre-treated with 2DG for 3 hours, ABT became a potent inducer of apoptosis, rapidly releasing cytochrome c from the mitochondria and activating caspases at submicromolar concentrations in a Bak/Bax-dependent manner. Bak is normally sequestered in complexes with Mcl-1 and Bcl-xL. 2DG primes cells by interfering with Bak-Mcl-1 association, making it easier for ABT to dissociate Bak from Bcl-xL, freeing Bak to induce apoptosis. A highly active glucose transporter and Bid, as an agent of the mitochondrial apoptotic signal amplification loop, are necessary for efficient apoptosis induction in this system. This combination treatment of cancer-bearing mice was very effective against tumor xenograft from hormone-independent highly metastasized chemo-resistant human prostate cancer cells, suggesting that the combination treatment may provide a safe and effective alternative to genotoxin-based cancer therapies.

## Introduction

2-Deoxy-D-glucose is a glucose molecule which has the 2-hydroxyl group replaced by hydrogen. 2DG is transported across the plasma membrane by a glucose transporter [1]. Once in the cytosol, 2DG is phosphorylated by hexokinase II and its product, 2-deoxyglucose 6-phosphate, is trapped in the cytosol and becomes an inhibitor of hexokinases, just as glucose becomes glucose 6-phosphate and becomes an inhibitor of hexokinases. However, as glucose 6-phosphate is hydrolyzed by glucose 6-phosphatase very rapidly, producing NADPH and generating energy, its counterpart, 2-deoxyglucose 6-phosphate, is a poorer substrate of glucose 6-phosphatase. Consequently 2-deoxyglucose 6-phosphate accumulates in the cytosol, inhibiting hexokinases and lowering cellular energy levels. It is estimated that the intracellular half-life of 2-deoxyglucose 6-phsophate is approximately 50 min in cancer cells [2]. Thus 2DG acts as an inhibitor of the glycolytic pathway.

The slower hydrolysis rates of 2-deoxyglucose 6-phsophate allow for glucose uptake to be measured in living animals by ^18^F-fluoro-2-deoxyglucose based Positron Emission Tomography (FDG-PET). Recent advances in FDG-PET has clearly shown much higher rates of glucose transporter activities in vivo, of almost all solid tumors compared to normal healthy tissues, confirming the Warburg Effect [1]. FDG-PET has also become a very sensitive way to detect tumors at a very early stage, e.g., early stage asymptomatic pancreatic cancer. Since 2DG accumulates predominantly in cancer cells and partially inhibits much-utilized glycolysis in these cells, administration of 2DG is a safe and effective way of slowing cancer growth [1]. However, the cytostatic activity of 2DG is generally short-lived, lasting only about a week [3]. Thus 2DG has been tried in combination with other chemotherapeutics, but again with mixed results. In some cases, it could lower the efficacy of other treatments [4]. One reason for decreased efficacy in these combination therapies might be that in some cells, 2DG activates an insulin-like growth factor receptor (IGFR), which could activate the PI3K-mTOR-AKT pro-survival pathway [5], [6].

Various drugs that target Bcl-2 and Mcl-1 have been tested for their abilities to induce apoptosis in cancer cells [7]. The most prominent among them are the Bcl-2 antagonists ABT-737 and ABT-263 [8], [9]. ABT-737 binds specifically and with high affinity to the Bcl-2 family of proteins, including Bcl-2, Bcl-xL and Bcl-w, but not to Mcl-1. ABT-737 was modified at three sites to create ABT-263 for improved oral bioavailability without the loss of affinity to the Bcl-2 family of proteins [9]. As a single agent, however, ABT-263/737 (ABT) induces apoptosis only in limited tumor types, such as lymphomas and some small cell lung carcinomas [8], [9], [10]. The reason for varied sensitivities to ABT is not entirely clear.

Apoptosis typically proceeds by either the intrinsic pathway, in which cytochrome c is released from mitochondria in a Bax/Bak-dependent manner, activating apoptosomes in the cytosol, or the extrinsic pathway, in which caspases are activated directly downstream of death receptors (reviewed by Salvesen & Riedl [11]). Often there is cross-talk between these two pathways. Because ABT binds mitochondrial proteins, ABT-induced apoptosis is thought to occur through the intrinsic pathway, but we do not know exactly how this is done.

The critical step in the intrinsic pathway of apoptosis is when cytochrome c is released from mitochondria because once cytochrome c is released into cytosol, it stimulates the formation of apoptosomes, the death executioner. For this reason, cytochrome c release is often called the point of no return [12]. Until several years ago, it was thought that calcium-activated mitochondria permeability transition pores (mPTP) may be required for cytochrome c release during the intrinsic pathway of apoptosis. However, it was shown that tBid-induced cytochrome c release and subsequent apoptosis could take place in the absence of VDAC [13] and cyclophilin D [14], [15], two essential components of the mPTP (reviewed in Yamaguchi & Perkins [16]). Instead, the prevailing dogma is that cytochrome c release occurs through mitochondrial outermembrane pores (MOMPs). Although MOMP is a well-established concept, its existence remains hypothetical because unlike nuclear pores or mPTP, there are no EM pictures of MOMPs. Likewise, MOMPs have not been isolated as a biological entity. Thus, it is not known whether Bak or Bax reside in MOMPs. The most detailed analysis of MOMP formation during apoptosis, came from studying lipid vesicles assembled with mitochondrial outermembrane proteins. In these studies, tBid-activated Bax was used to stimulate MOMP assembly on the vesicles [17], stimulating the release of proteins as large as 2000 kDa [18] through pores with diameters of 25–100 nm [19]. If MOMPs on mitochondria are about the same size, one would expect to see them using electron microscopy (EM), but they haven't been imaged. Thus, the existence of MOMPs remains hypothetical. Nevertheless, from a mechanistic viewpoint, the existence of MOMPs comprised either wholly or in part by Bax and Bak makes sense.

BH3-only proteins such as tBid, BimS are called “activators” because they directly interact with Bax or Bak, changing their conformation and inducing oligomerization, in other words “activating” them, leading to cytochrome c release and apoptosis. More recent findings, however, suggest another scenario in which apoptosis takes place in the absence of activator proteins. First, Huang and colleagues showed that Bak is normally sequestered by Mcl-1 and Bcl-xL, and Bak must be released from both before it can form oligomers [20]. The idea that inactivating some anti-apoptotic proteins without the help from some activator can induce apoptosis, is not without controversy [21], [22]. We favor the idea because Huang and colleagues found that inactivating both Bcl-xL and Mcl-1 was enough to induce apoptosis in Bid and Bim knockout MEFs [23], and Hayashi and colleagues found the same in Bid and Bim deficient platelet cells [24].

Assuming that Bim and Bid are not involved in ABT-induced apoptosis, we can speculate reasons for varied sensitivities to ABT-737. Since ABT-737 binds to Bcl-xL, inactivating it, but it does not bind to Mcl-1, if there are less Mcl-1 proteins present, then the efficacy of ABT-737 must increase. Indeed, Huang and colleagues found that by depleting Mcl-1 from HeLa cells with siRNA, they became sensitive to ABT-737 [25]. Many groups also reported inverse correlations between cancer cell sensitivity to ABT-737 and Mcl-1 expression levels in many cancer types (see Information S1). However, there are exceptions; HL-60 human promyerlocytic leukemia cells express reasonably large amounts of Mcl-1, but they are nevertheless, sensitive to ABT-737 [26]. Thus how ABT-737 induces apoptosis in some cancer cells and not in others, remains poorly understood.

We initiated this project in order to search for agents that can increase the efficacy of ABT. When we co-treated human tumor cell lines with ABT and drugs that interfere with cancer specific cellular metabolism, we found that 2DG pre-treatment vastly improved the efficacy of ABT without harming healthy cells. We show that 2DG-ABT induces apoptosis without altering Mcl-1 levels in ABT-resistant cells. When cancer cells resistant to ABT are pre-treated with 2DG, the protein levels of Mcl-1 remain the same, but Bak-Mcl-1 association is lost, sensitizing cells for ABT-induced apoptosis. How 2DG treatment of cancer cells disrupts Bak-Mcl-1 is not known. Thus, cancer cell sensitivity to ABT seems to be determined not only by the protein levels of Mcl-1, but also by how much Bak is sequestered by its association with Mcl-1.

## Results

### 2DG-ABT efficiently induces apoptosis of cancer cells

**Figure 1:**
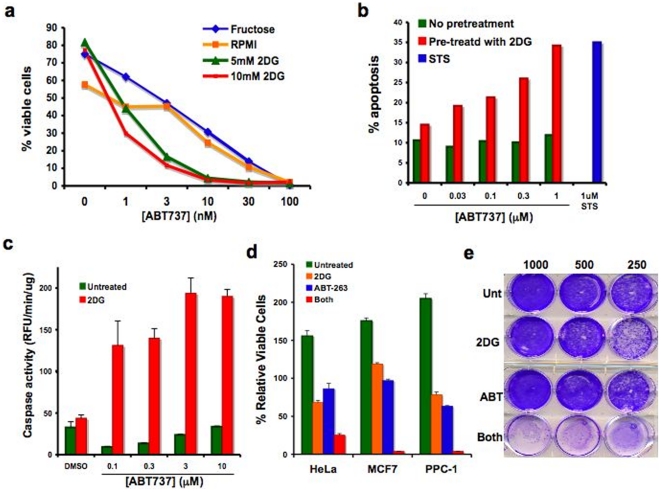
Annexin V-staining of 2DG-ABT treated RS(4;11) and HeLa cells. **a,** RS(4;11) cells pre-treated with 20 mM fructose or 0–10 mM 2-deoxy-D-glucose in regular RPMI medium containing 12 mM glucose, for 1 hour before the addition of ABT-737 at indicated concentrations. 9 hours later, cells were analyzed by FACS for Annexin-V positivity. Chi Square Test of Independence for two variables, 2DG and ABT, was P(x~_1_^2^>193.35) = 0.000000. Since the combination was clearly better than expected values, the statistic suggests synergistic interaction between 2DG and ABT-737. **b,** HeLa cells were pre-treated for 1 hour either with or without 10 mM deoxyglucose in DMEM containing 12 mM glucose, before adding ABT-737. Six hours later, cells were analyzed by FACS for Annexin-V staining. **c,** HeLa cells were pre-treated for 3 hours with 2DG before the addition of ABT-737. Cells were assayed for caspase activity at 3 hours after ABT-737 addition. **d,** HeLa, PPC-1 and MCF-7 cells were treated with 10 mM 2DG for 3 hours before adding 1 µM ABT-263 for overnight incubation. Trypan blue-negative (viable) cells were counted and graphed (mean ± std dev; n = 3). **e, Combination therapy reduces clonogenic survival of PPC-1 cells.** PPC-1 cells were treated just once for 24 hrs with 5 mM 2DG, 1 µM ABT-263, both, or were left untreated. 250–1000 cells were plated per 30 mm dish, and 12 days later, surviving cell colonies were stained.

Applied as a single agent, 30 nM of ABT-737 killed 90% of RS(4;11) non-Hodgkin's B-cell lymphoma cells in 9 hours (Fig. 1a). However, a 1-hour pre-incubation of RS(4;11) cells with 5 to 10 mM 2DG in medium containing 10 mM glucose increased the efficiency of ABT dramatically, killing 90% of cells with just 3 nM ABT-737. 2DG alone did not cause cell death during the 10-hour incubation. When we tested for caspase activation, it was found that as little as 1 nM of ABT-737 could activate caspases in 3 hours as long as the cells were pre-incubated with 2DG for 3 hours. No caspase activation beyond the background was observed in cells that were incubated with 2DG alone. Thus, it can be concluded that 2DG sensitizes RS(4;11) cells to ABT-induced apoptosis. We repeated the experiment using various concentrations of ABT-263 and counted cells by trypan-blue dye to score for dead cells 24 hours after ABT addition (Fig. S1a). The result also showed a synergistic effect of 2DG and ABT.

Next, we tested whether 2DG could sensitize ABT-resistant tumor cells to ABT-737 or ABT-263. Initial experiments were performed using HeLa cervical cancer cells. HeLa cells were pre-treated for 1 hour with 2DG before the addition of ABT-737. Nine hours after the addition of 1 µM of ABT-737, approximately 35% of HeLa cells that were pre-treated with 2DG, were Annexin V-positive (Fig. 1b). This rate of cell death was comparable to that seen after the treatment of HeLa cells with 1 µM staurosporine (STS), a well-known inducer of apoptosis in HeLa cells In another set of experiments, when HeLa cells were pre-treated for 3 hours with 10 mM 2DG in a medium containing 25 mM glucose, robust caspase activation was observed within 3 hours at concentrations of ABT-737 as low as 0.1 µM (Fig. 1c). Without 2DG pre-treatment, even 10 µM ABT-737 could not activate caspases during the same period. This experiment was also repeated using ABT-263 and scoring dead cells with trypan-blue dye incorporation (Fig. S1b). Again, it showed a synergistic effect of ABT-263 and 2DG. ABT-737 and ABT-263 were compared side-by-side using three-hour 2DG-pretreatment of HeLa cells. ABT-263 was slightly better than ABT-737 in inducing apoptosis (Fig. S2a).

### Conditions for 2DG-ABT induced apoptosis

Kinetic and dose-response studies with HeLa cells showed the following: (i) the presence of glucose in the medium is necessary for 2DG-ABT induced apoptosis (Fig. S2b), and the optimal molar ratio for apoptosis induction with 2DG:glucose is approximately 0.4 to 1.0; (ii) 2DG should be added 3 to 4 hours prior to the addition of ABT (Fig. S2c & d); and (iii) caspase activation is observed within 3 hours of ABT addition. These conditions may reflect the stability of ABT-263/737 in solution (approximately 4 hours [9]), as well as the nature of intercellular signals generated by 2DG; it must take 3 to 4 hours for 2DG to generate a strong enough signal to be effective. Under these conditions, the reduction in cellular ATP concentration is very slight (Fig. 5a), suggesting that ATP insufficiency does not provide an explanation for the improved sensitivity to ABT-737 and ABT-263.

All subsequent experiments were performed using the following conditions: Add 5–10 mM 2DG to cell culture. Three hours later, add 1–3 µM ABT. Assay for caspase activity and cytochrome c release from mitochondria three hours after ABT-treatment. Six hours after ABT-treatment, assay for Annexin V staining. 24 hours later, count viable cells by trypan blue dye exclusion assay. The response to the protocol varied from cell line to cell line. Among cancer cells that responded well were breast cancer cell line MCF-7 and hormone resistant prostate cancer cell line PPC-1. In both cases, over 95% of cells were eliminated in 24 hours by the combination treatment (Fig. 1d). A single agent application of 2DG caused some death (31.3% for HeLa, none observed in MCF-7, and 21.5% for PPC-1). Since all cells have varied amounts of glycogen as stored source of glucose, the rates of cell death by 2DG was largely dependent on the previous history of cultured conditions. Cultured cells normally overcome 2DG-induced growth-arrest and start to grow in 24–48 hours (data not shown). Furthermore, when 2DG was injected into cancer-bearing mice, it did not cause tumor regression, but delayed tumor growth by 5–6 days. After this short delay, tumors started to grow. Other groups had made similar observations [4]. The reason for the acquired resistance to 2DG is not known. As a single agent, the effect of ABT-263 was also marginal (13.7% for HeLa, 3.1% for MCF-7 and 36.7% for PPC-1). The effects of 2DG-ABT combination far exceeded the additive effects of 2DG and ABT in all three cell lines (HeLa expected viable cells 59%, observed 25%, MCF7 expected viable cells 115%, observed 3.8%, and PPC1 expected viable cells 49.7%, observed 3.9%). The Clonogenic Cell Survival assay also showed that over 95% of PPC-1 cells were eliminated by just one treatment while single agent applications of ABT or 2DG were largely ineffective (Fig. 1e). Given that p53 is inactive in some of the highly responsive tumor lines including PPC-1 [27], [28], [29], we conclude that the combination-induced apoptosis is p53-independent. The possible reasons for varied responses will be discussed later.

In order to understand exactly what molecular events take place when ABT is added to 2DG treated cells, we decided first to investigate what type of apoptosis is being induced by the combination of 2DG and ABT.

### Combination treatment with 2DG and ABT induces apoptosis through the intrinsic pathway

**Figure 2:**
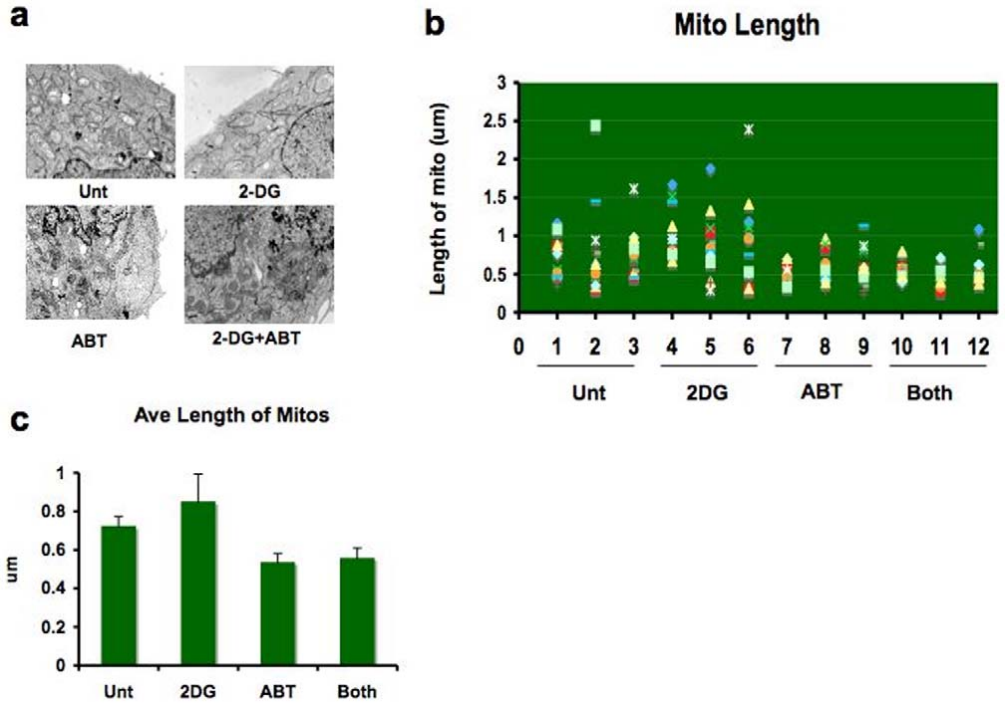
Combination of 2DG and ABT-263 does not cause mitochondrial outer membrane rupture. HeLa cells treated with either 10 mM 2DG for 6 hours, 1 µM ABT-263 for 3 hours, or 2DG for 3 hours before adding ABT-263 for an additional 3 hours, or left untreated. **a,** Cells were fixed for electron microscopy (see Method). Three cells from each treatment group were examined closely for the length of mitochondria. We did not observe a single instance of ruptured mitochondrial outermembranes. **b,** From each cell, a minimum of 12 mitochondria were randomly chosen for measurement. **c,** Average lengths of mitochondria became shorter with treatments containing ABT-263 (mean +/− std dev ). Since ABT-263/737 binds to Bcl-2 family members, which are known to be involved in mitochondrial fusion/fission, ABT may have altered the balance of mitochondrial fusion/fission to the fission side (p = 0.0001, 95% confidence interval of this difference: from 14.5407 to 37.2513; unpaired t-test).

It has been reported that the application of 10 nM ABT-737 for just 2 hours causes the mitochondrial outer membrane to rupture in chronic lymphocytic leukemia (CLL) cells [30]. This is not a normal phenomenon in apoptosis. Cytochrome c is usually released through mitochondrial outermembrane pores in orderly fashion [31]. The destruction of mitochondria takes place long after cytochrome c release by the activities of fully activated cell death machineries. When we examined HeLa cells treated with the 2DG-ABT combination using EM, rupture of mitochondrial outer membranes was not detected (Fig. 2a). In ABT-treated cells, however, slight shortening of mitochondria were observed (Fig. 2b&c); from 0.717+/−0.05 µm for untreated to 0.530+/−0.05 µm for ABT only, 0.553+/−0.06 µm for 2DG+ABT, and slight lengthening of mitochondria in 2DG-treated cells at 0.844+/−0.05 µm. We speculate that since Bcl-xL and Bcl-w are known to be involved in mitochondrial fusion/fission [32], ABT may have inhibited their activities. As for the lengthening of mitochondria under 2DG, it may have created increased demand for mitochondrial respiration, and to meet that burden, mitochondria may have become longer. Even though 2DG and ABT had opposite effects on the length of mitochondria, they both influenced the physiologies of mitochondria. Even though some speculates that mitochondria fusion/fission machinery may also play a role in apoptosis, the idea remains controversial [33].

**Figure 3:**
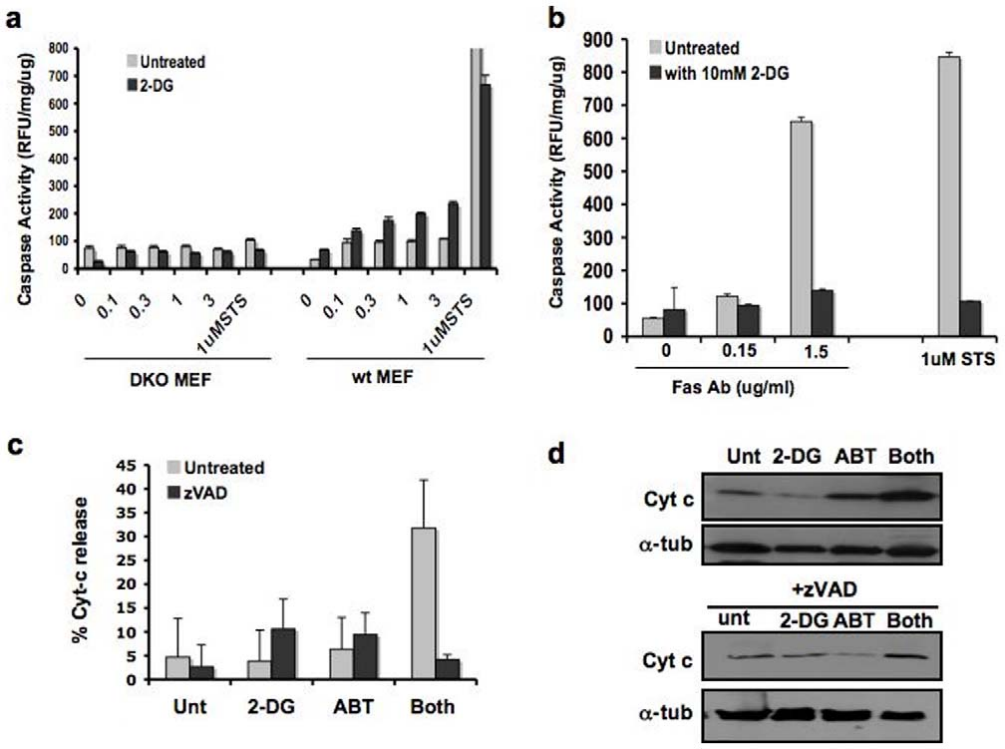
2DG-ABT induced apoptosis is a non-canonical mitochondria-dependent form of apoptosis. **a,** Wt, and Bax/Bak DKO MEFs were treated with 10 mM 2DG for 3 hours before the addition of 3 µM ABT-263 for 3 hours. 3 hours later, cells were harvested for caspase activity assays. (See also Fig. S5a). **b,** HT1080 cells were treated with or without 2DG for 3 hours. Then cells were treated with either 1 µM STS or anti-Fas (CD95) antibody at indicated concentrations (µg/ml). Cells were harvested 3 hours after, and assayed for caspase activity. Interestingly, 2DG seems to have interfered with Fas-induced apoptosis. In repeated experiments, we observed consistently lower rates of caspase activation in 2DG pre-treated cells, but the degrees of inhibitory activity varied from experiment to experiment. We are not sure of its cause. **c,** For the first 3 hours, HeLa cells were either treated with 10 mM 2DG or left untreated and then treated with 10 µM z-VAD, 1 µM ABT-737, both, or neither for 3 more hours. The cells were fixed and stained for DNA (red) and cytochrome c (green) and examined for loss of cytochrome c staining, as described in Methods. Bar indicates 100 µm. The representative images are shown in Fig. S3. This graph shows the percentage of cells with loss of cytochrome c staining. The combination treatment had higher rates of cells with released cytochrome c (p = 0.008 by unpaired t-test ). **d,** Cells treated as in **c** were fractionated and cytosolic fractions were analyzed by western blots.

To determine if 2DG-ABT-induced apoptosis is through the intrinsic pathway, we used wild-type (wt) mouse embryo fibroblasts (MEFs) and Bax/Bak double knockout (DKO) MEFs. These DKO cells are lacking the intrinsic pathway. In wild-type MEFs pre-treated with 2DG, caspase activation was induced within 3 hours of ABT addition (Fig. 3a). However, there was no rapid caspase activation in Bak/Bax DKO MEFs. Therefore, we concluded that 2DG-ABT-induced apoptosis occurs by the Bak/Bax-dependent intrinsic pathway (see also Fig. S5a).

To test whether 2DG also sensitizes tumor cells to the extrinsic pathway of apoptosis, we used HT1080 cells, in which there is no cross talk between the intrinsic and extrinsic pathways [34]. Pre-treatment of HT1080 cells with 2DG for 3 hours did not enhance Fas-induced apoptosis but lowered caspase activation (Fig. 3b). Thus, 2DG did not enhance the extrinsic pathway.

### 2DG-ABT induces cytochrome c release through a caspase-dependent mechanism

A critical step in the intrinsic pathway is the release of cytochrome c from mitochondria. When 1 µM of ABT-737 was applied to HeLa cells that had been pre-treated with 2DG for 3 hours, we observed the release of cytochrome c in over 30% of the cells within the next 3 hours but not in untreated cells or cells treated with either 2DG or ABT alone (Fig. 3c and Fig. S3).

In canonical mitochondria-dependent forms of apoptosis such as staurosporine-, etoposide- or UV-induced cell death, the release of cytochrome c from the mitochondria occurs upstream of caspase activation and can proceed in the presence of caspase inhibitors [35], [36]. By contrast, during death receptor-induced apoptosis, caspases activated by these receptors can also cleave the Bid protein, producing truncated Bid (tBid), with tBid then inducing Bak/Bax oligomerization and the formation of pores at the mitochondrial outer membranes through which cytochrome c escapes. Thus, a pan-caspase inhibitor such as z-VAD blocks death receptor-induced cytochrome c release from mitochondria. We found that 2DG-ABT induced cytochrome c release was also blocked by z-VAD when added synchronously with ABT (Fig. 3c & d, Fig. S2), yet we showed that the 2DG-ABT induced apoptosis is Bax/Bak-dependent. This conundrum could be explained by a Bid-dependent amplification loop in which a small amount of cytochrome c released by ABT could activate a cascade of caspases (such as caspases 9,3,6 and 8) in the cytosol, cleaving Bid to generate tBid, with tBid in turn activating Bak/Bax and causing further release of cytochrome c from the mitochondria [37], [38].

### The effects of Bid depletion on 2DG-ABT-induced apoptosis

**Figure 4:**
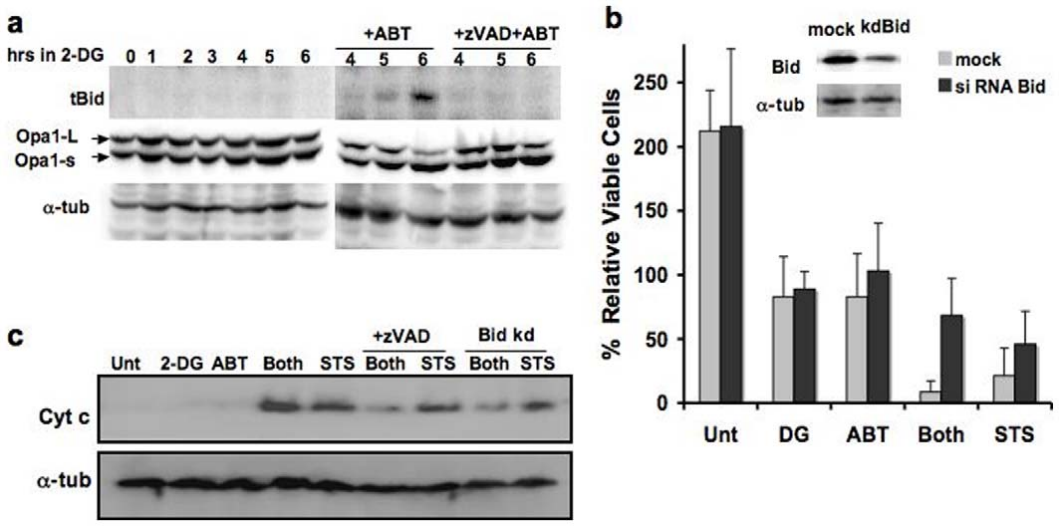
The Bid-based feedback loop is required for efficient 2DG-ABT induced apoptosis. **a, Bid is activated during 2DG-ABT induced apoptosis.** Western blots of PPC-1 cell lysates treated with 10 mM 2DG for 0–6 hours (left panel), with either 1 µM ABT-263 alone or ABT-263+10 µM z-VAD for the last 3 hours (right panel), probed with the anti-tBid antibody (upper panels), anti-Opa1 antibody (middle panels), and anti-alpha-tubulins (lower panels). Note: the loss of Opa-1-L was used as a marker for remodeled mitochondrial cristae during cytochrome c release from the mitochondria [34]. **b, The effects of Bid depletion on 2DG-AB-induced apoptosis.** Insert: western blots of PPC-1 cell lysates, mock transfected (left lane) or transfected with Bid siRNA (right lane). Bid (+) and Bid (−) PPC-1 cells were treated with either 2DG, ABT-263 (1 µM), both, 10 µM ABT-263, or left untreated for 24 hours. The viable cells were counted and the increase/decrease over the input numbers were graphed. **c,** In PPC-1 cells either pre-treated with 2DG for 3 hours or without pre-treatment, then 1 µM ABT-263 or 1 µM STS was added for three hours, and cytosolic fractions were analyzed by western blots. In parallel experiments, Bid-depleted PPC-1 cells were treated with the combination of 2DG and ABT-263 or with STS and analyzed (designated as Bid kd, last 2 lanes). Cells were harvested 3 hour after ABT/STS treatment, and cytosolic fractions were analyzed with western blots.

We explored whether the hypothesized Bid-based amplification loop plays a role in 2DG-ABT-induced apoptosis. First, we determined whether Bid is cleaved during 2DG-ABT induced apoptosis. In both HeLa and PPC-1 cells, 2DG pre-treatment followed by the addition of 1 µM ABT-263/737 induced cytochrome c release from the mitochondria in 3 hours. In both cell types, the release of cytochrome c was blocked by z-VAD-fmk. With the PPC-1 line, almost 100% of the cells released cytochrome c, causing nearly 100% apoptosis. With HeLa cells, the proportion of cells displaying cytochrome c release within the first three hours was approximately 30% (Fig. 3c). Truncated tBid was seen 3 hours after the addition of ABT in PPC-1 and HeLa cells (Fig. 4a, data not shown). tBid was absent in cells treated with 2DG alone. When z-VAD-fmk was added with ABT, the cleavage of Bid was blocked.

To determine what effect, if any, Bid has on 2DG-ABT-induced apoptosis, we depleted the Bid protein by siRNA. 2DG-ABT-induced apoptosis was largely blocked in Bid-depleted PPC-1 cells. Bid depletion increased the percentages of viable cells by ∼8-fold (p<0.0077; unpaired t-test) (Fig. 4b). The amounts of released cytochrome c correlated with apoptotic rates (Fig. 4c). Thus, the Bid-dependent amplification loop is required for efficient 2DG-ABT induced apoptosis. Bid depletion had a similar impact on apoptosis induced by 10 µM ABT-263 but the impact was less on STS-induced apoptosis (data not shown and Fig. 4b & c). Lastly, Bid KO MEFs also displayed reduced sensitivity to 2DG-ABT treatments (Fig. S5a). We concluded that Bid plays a significant role in 2DG-ABT-induced apoptosis.

### Search for 2DG effectors

Since 2DG-ABT combination-induced apoptosis is Bax/Bak-dependent, we examined the effects of 2DG on Bax and Bak during the three-hour pre-incubation. Some cells cultured under hypoxic conditions or in glucose-depleted media for extended periods, become sensitized to apoptosis [39], [40], [41]. Although no universal marker exists for cellular sensitivity to apoptosis, Bax is known to translocate to the mitochondria prior to apoptosis and necrosis in some cases [41]. Bax translocation also seems to be a distinct step, and not necessarily concurrent with its activation [42]. However, in HeLa cells treated with 2DG alone, Bax translocation was not induced for at least 16 hours (Fig. S4a). Thus, unlike in cells cultured in glucose-depleted media, Bax translocation does not take place during the 2DG pre-incubation period. Furthermore Bax knockout MEFs responded to the 2DG-ABT-combination treatment (Fig. S5a), suggesting that Bax may not play a critical role in priming of cancer cells by 2DG. Of course, in the absence of Bak, Bax is already be at mitochondria, playing the role of Bak, and Bax may be activated by a different mechanism when it is at mitochondria [43].

**Figure 5:**
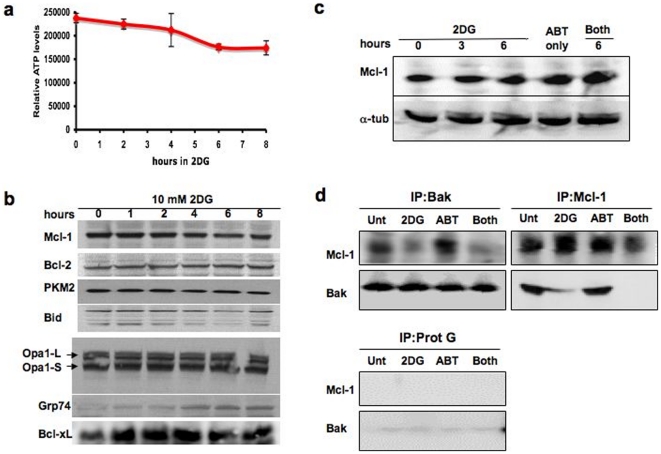
2DG disrupts Bak-Mcl-1 association, priming cancer cells without lowering protein levels of Mcl-1. **a, The effect of 2DG on cellular ATP levels.** The amounts of ATP were measured during HeLa cell culture with 10 mM 2DG in the presence of 25 mM glucose in DMEM supplemented with 10% serum.** b, Protein profiles during 2DG incubation of HeLa cells.** Samples were taken from HeLa cells treated as in **a**, and various protein contents of pro- and anti-apoptotic proteins were analyzed by Western blots. In this experiment, we see increases in Bcl-xL for the first hour. When we repeated the experiment twice, Bcl-xL levels remained the same.** c, Mcl-1 levels remained unchanged during 2DG-ABT-induced apoptosis.** PPC-1 cells were treated with 10 mM 2DG for 6 hours, 1 µM ABT-263 for 3 hours, or 10 mM 2DG for 6 hours of which the last 3 hours were incubated with 1 µM ABT-263.** d, Bak and Mcl-1 do not co-precipitate in 2DG treated cells.** PPC-1 cells were treated as in **c**. Bak and Mcl-1 were immunoprecipitated by specific antibodies conjugated to Protein G-Sepharose and the co-precipitated proteins were analyzed by Western blots.** Left Upper Panels**, Bak co-precipitates, **Right Upper Panels**, Mcl-1 co-precipitates, and **Left Lower Panels**, Protein G-Sepharose precipitates. For technical reasons, we could not immunoprecipitate Bcl-xL and analyze its content.

Because 2DG can affect glycosylation, 2DG-treatment might induce ER stress. Indeed, expression of the ER stress marker, Grp74, was observed in HeLa cells treated with 2DG for 2 to 4 hours (Fig. 5b and Fig. S4b). We hypothesized that if 2DG is acting through ER stress, we might be able to sensitize HeLa cells for ABT-induced apoptosis by pre-treating them with other ER stress inducers such as thapsigargin. However, this was not the case (Fig. S4c). Thus, 2DG appears to sensitize tumor cells to Bcl-2 antagonists through mechanisms that do not depend on glucose-depletion or ER stress.

### 2DG disrupts Bak-Mcl-1 association, priming cells for ABT-induced apoptosis

It has been reported that culturing cells in glucose-depleted medium or in the presence of 2DG for extended time (24 hours or longer), could lower cellular ATP concentrations and prime various cells for apoptosis [39], [40], [44]. During this induced ATP insufficiency, translation of anti-apoptotic Mcl-1 is hampered, lowering protein levels of Mcl-1 and priming cells for apoptosis. We tested whether 2DG lowers Mcl-1 protein levels. As mentioned earlier, the decreases in cellular ATP concentration is very slight during the first 8 hours with 2DG (Fig. 5a). Furthermore, no significant changes were observed in the levels of Mcl-1 and several other anti- and pro-apoptotic proteins during the first eight hours of 2DG exposure (Fig. 5b). The levels of Mcl-1 also remained unchanged after ABT addition in HeLa, PPC-1 and other cells (Fig. 5c and Fig. S4d). Altogether, these experiments suggest that ABT-263/737 induces apoptosis without depleting the Mcl-1 protein. We cannot exclude the possibility that more subtle changes such as phosphorylation or other post-translational modifications of Mcl-1 and other anti-apoptotic proteins occurred during 2DG-incubation, which may be responsible for the sensitizing effect of 2DG. Indeed, we found that even though Mcl-1 protein levels seem unchanged, Mcl-1-Bak association was lost during 2DG pre-incubation, and remained absent during the subsequent incubation with 2DG and ABT (Fig. 5d). Since both Mcl-1 and Bcl-xL can sequester Bak from forming oligomers, loss of Mcl-1-Bak association during 2DG incubation can sensitize cells because ABT can now effectively bind Bcl-xL and free Bak. Earlier we noted that HL-60 is sensitive to ABT-737 despite having large amounts of Mcl-1 expressed. It was also shown that anti-Bak antibody failed to co-precipitate Mcl-1 even though it can co-precipitate Bcl-2 [26]. Thus the reason for its sensitivity might be the absence of Bak-Mcl-1 complex. We do not know why Bak does not bind to Mcl-1 in HL-60 cells. Currently we are investigating the possible molecular changes to Bcl-xL and Mcl-1 disruptive to their association.

**Figure 6:**
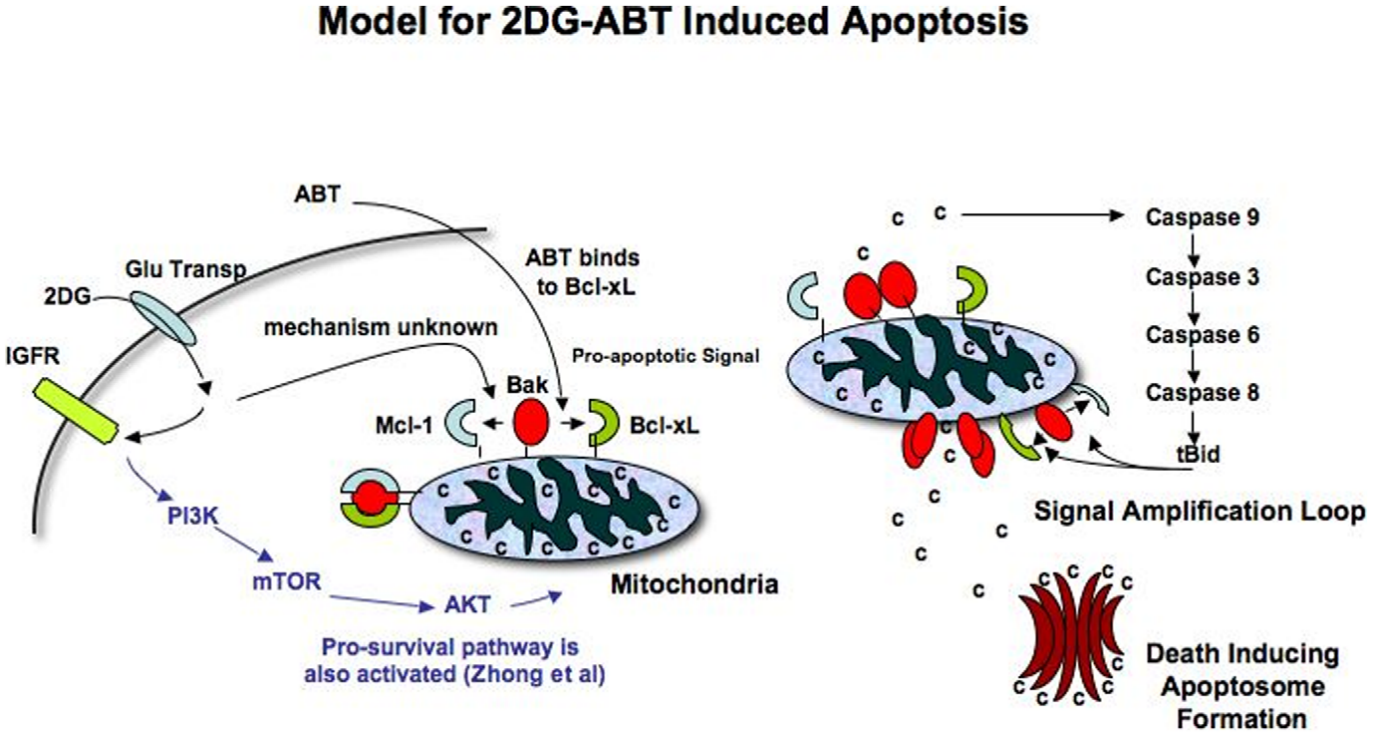
Model of 2DG-ABT Induced Apoptosis. **a, 2DG **is specifically taken up by cancer cells through a glucose transporter. Once inside, it is phosphorylated and becomes a hexokinase inhibitor. At the same time, it activates several signal transduction cascades. One of them is IGFR-PI3K-mTOR-AKT pro-survival pathway. Through another cascade activation, Bak-Mcl-1 association is lost. When ABT is added 3 hours later, it can bind to Bcl-xL and disrupts its association with Bak. **b,** Freed Bak oligomerizes, releasing a small amount of cytochrome c into the cytosol. It activates cascades of caspases 9-3-6-8 and caspase 8 cleaves Bid, generating BH3 only tBid protein. Since tBid can dissociate Bak-Mcl-1 and Bak-Bcl-xL association, it induces mitochondria outermembrane pore formation through which more cytochrome can be released. Released cytochrome c recruits Apaf1, caspase 9 and dATP, forming a “death executioner” apotosome complex.

Taken together, we propose the following model for 2DG-ABT induced apoptosis in cancer cells (Fig. 6): 2DG induces changes in Bak or Mcl-1 so that they can no longer associate. ABT binds to the BH3 pocket of Bcl-xL, breaking its hold on Bak. Released Bak forms oligomers, releasing a small amount of cytochrome c. Released cytochrome c now activates caspase cascades, generating tBid which can induce full-scale outer membrane pore formation on mitochondria. A large-scale release of cytochrome c triggers full-scale apoptosome formation and cell death. This model is supported by our findings.

### 2DG activates PI3K and generates a pro-survival signal

It has been reported that 2DG can activate multiple signal transduction cascades, activating well-known kinases such as PI3K, MAPK and IKK that phosphorylate over 30 proteins, some of which are known to be involved in apoptosis [5], [6]. Thus it is possible that one of these signals is responsible for the loss of Bak-Mcl-1 association. However, these reports caution that 2DG can also activate a pro-survival pathway via PI3K and the phosphorylation of AKT; thus, adding 2DG could hinder, rather than facilitate apoptosis, depending on the cell type. Indeed, we have consistently observed lower rates of STS-induced apoptosis when cells were pre-incubated with 2DG (Fig. 3a and Fig. S2c). We thus tested whether pro-survival signals are generated in HeLa cells during 2DG-ABT induced apoptosis by using LY294002, a PI3K inhibitor, to ascertain if we could block this signal. With the addition of LY294002, an almost three-fold increase in caspase activities was recorded in 2DG-ABT treated HeLa cells (Fig. S5b), confirming that 2DG generates a pro-survival signal through PI3K in HeLa cells. 2DG also induced phosphorylation of ATK in HeLa cells (data not shown). Thus, 2DG seems to be sending complicated and often conflicting signals (Fig. 6). The way in which cells respond to 2DG might determine if they will become sensitized or resistant to apoptosis. We thus decided to examine how commonly available cell lines and primary cells would respond to the 2DG-ABT combination treatment.

### Most cancer cells responded to the 2DG-ABT treatment

Most cancer cells tested responded well to the combination treatment; apoptosis was induced in most cells with just 1 µM ABT-263/737. The list of 2DG-ABT sensitive cells includes cells from leukemia (RS(4;11), BP3), cervical cancer (HeLa), prostate cancer (PPC-1/PC-3), hepatocarcinoma (HepG2), breast cancer (MCF-7), and most small lung carcinomas. However, cells with low glucose transporter activities such as confluent NIH3T3 cells and CLL cells that do not grow in culture could not be sensitized by 2DG for ABT-induced apoptosis. Small-cell lung carcinoma-derived cell lines, on the other hand, grow well in culture, and most of them are sensitive to ABT [8], [45]. The NIC H82 cell line is a well-studied exception to this rule, but why it is resistant to ABT remains unclear [8], [46]. However, we now know that activation of the Bid protein is necessary for efficient apoptosis induction by ABT, and the CASP8 gene encoding the Bid-activating capsase-8 enzyme is deleted in NCI H82 cells [47], [48]. Thus, it seems likely that the absence of caspase-8 makes NCI H82 cells resistant to ABT-induced apoptosis.

Pancreatic cancer cells are highly resistant to apoptosis. Despite our repeated attempts at inducing apoptosis in PANC-1 pancreatic cancer cells, the 2DG-ABT treatment failed to induce apoptosis. PANC-1 cells express Bid, Bcl-2 and Bak, and cleaved Bid is observed in TNF-treated cells, and these cells have active glucose transporter(s) - all the conditions necessary for 2DG-ABT induced apoptosis. Thus, these conditions are clearly not sufficient. We are trying to discover why these cells are so highly resistant. We are also setting up screens to identify apoptosis inducers specific for 2DG-treated pancreatic cancer cells.

### 2DG improves anti-tumor activity of ABT-263 in a xenograft model of human prostate cancer

Previous work demonstrated that either 1 g/kg of 2DG [49], [50] or 100 mg/kg of ABT-263 [9] could safely be injected into mice. To test the safety of the combination, we treated wild-type C57BL/6 mice with intraperitoneal injection of 0.5 g/kg of 2DG+/−0.5 g/kg of glucose in the morning, and 100 mg/kg of ABT-263 in the afternoon. The combination treatment produced only a few side effects, including mild lymphopenia and reversible thrombopenia, similar to ABT-263 alone (data not shown) [9], [51], [52].

**Figure 7:**
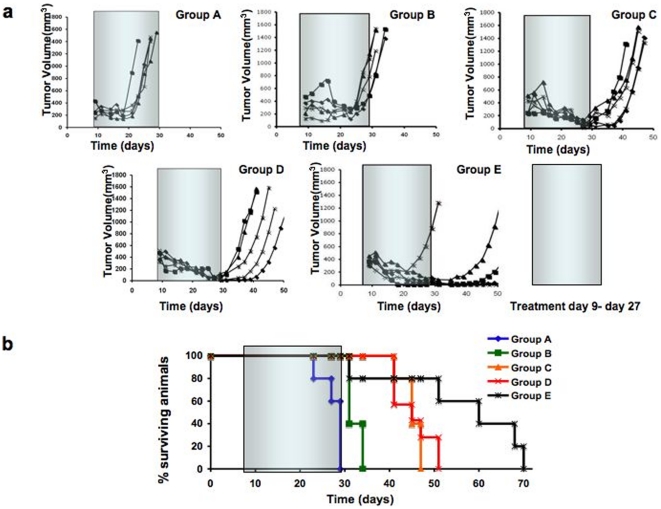
Treatment of tumor-bearing mice with 2DG-ABT combination. Five million PPC-1 human prostate cancer cells were xenografted onto nude mice as described in the text and the method. **a,** Mice Tumor volumes were measured in mice (n = 7 per group) treated with diluent only (group A), 2DG (group B), ABT-263 (group C), 2DG plus ABT-263 (group D), or 2DG mixed with D-glucose plus ABT-263 (group E), as explained in the text, where drugs were administered 3 times weekly between day 9 and 27 (gray rectangles). **b,** Survival of tumor-bearing mice was determined at various times post-inoculation with PPC-1 cells. The % of surviving mice is indicated from groups A-E (n = 7 per group). The difference of survival days between Group C & D versus Group E is p = 0.0283, 95% confidence interval of this difference: from −20.98 to −1.36 by unpaired t-test.

For xenograft implants, we chose a highly chemorefractory tumor line PPC-1 [27], [28], [29], [53], [54], [55], [56]. Five million PPC-1 cells were injected into each athymic nude mouse, and the mice were treated three times a week for 3 weeks from day 9 to day 27 with the following regimens: Group A mice received buffer only, Group B mice received 2DG only, Group C mice received ABT-263 only, Group D mice received 2DG in the morning and ABT-263 in the afternoon, and Group E mice received a glucose/deoxyglucose mix in the morning and ABT-263 in the afternoon. After treatment with 2DG only, there was only a 5- to 6-day delay in tumor growth (Fig. 7a). In contrast, except for one mouse in Group E, the tumors in all of the mice in Groups C, D, and E regressed during treatment. The rates of tumor regression among these groups were roughly similar. However, when treatments were concluded at day 27, tumors began to grow in mice in Groups C and D, such that all these mice required euthanasia before day 50. The mice in Group E fared better, and most of their tumors remained small until day 50; these mice survived an average of 2 weeks longer than the mice in Groups C and D (Fig. 7b). The mouse experiments were repeated twice with very similar results (Fig. S6). These results concur with our tissue culture results, demonstrating the clear advantage of the combination treatment. When we surveyed literature on treatments of PPC-1/PC-3 tumor bearing mice, we only found treatments that had arrested or slowed tumor growth (cytostatic effects) – and we alone had the treatment to induce tumor regression (cytoxic effect) [27], [55], [56], [57], [58], [59]. To summarize, these preclinical data suggest that deoxyglucose-ABT-263/737 combination therapy may provide a safe and effective alternative to the genotoxin-based chemotherapies currently used for treatment of cancer.

## Discussion

In order to induce apoptosis in cancer cells, genotoxin-based therapies activate cell cycle checkpoint and genomic surveillance programs. Rapidly dividing cancer cells are also under pressure to compromise those same programs. As cancer progresses and accumulates more mutations, it seems natural that it becomes more resistant to genotoxin-based therapies. Therefore, to effectively treat advanced cancers, we need a different approach.

Uncontrolled cell growth is one of the defining features of cancer. Indeed, most cancer cells have multiple mutations in the components of growth regulatory machineries. By targeting highly activated growth-signaling pathways, we could not only arrest the cell growth, but also induce apoptosis in cancer cells. In fact, the oncogenic addiction paradigm postulates that cancer cells rely on just one oncogene to tip the balance between the induction of rapid cell cycle and the activation of cell cycle checkpoint that induces apoptosis [60], [61], [62]. Based on the oncogene addiction paradigm, targeted therapies deploy specific inhibitors of the presumed key oncogenes to treat particular cancer types [63]. The oncogenic addiction concept gained credence when Imatinib (Imatinib mesylate; commercial name Gleevec, originally STI571), a specific inhibitor of bcr-abl protein expressed in chronic myeloid leukemia (CML), successfully treated CML, achieving 5-year incident-free survival rates around 90% in most studies [64], [65]. Furthermore, because inhibitory effects of Imatinib are limited to a few kinases, Imatinib had shown few side effects.

Since the development of Imatinib, many more targeted drugs have been tested in clinical trials. These trials showed that many promising drugs induced rapid tumor regression with little side effects, resulting in near complete cancer remission within a few weeks. However, all too often, cancer returned within a year in treatment-resistant forms [66]. There were two ways that cancer gained resistance to the treatment: 1) mutation in the targeted oncogene and 2) gain of function mutations on the downstream effectors that constitutively activate them [67], [68], [69]. These mutations may have been in a small number of cells in the initial tumor mass, or they may have developed during the pro-longed treatment. Trying to remedy the situation, more targeted drugs were developed in some cases [70].

With knowledge gained through clinical trials, the oncogene addiction model was revised [71]; if we look at carcinogenesis as a step-by-step mutational process, oncogene addiction must occur after the critical oncogene activation and before the constitutively activating mutations of its down-stream effectors. Of course, during cancer development, there could be more than one oncogene addiction phase. In a later phase, cancer cells may depend on a different oncogene for cell growth and survival. Thus there may be another opportunity later on to treat cancer with a different targeted drug.

Since tumors tend to be a heterogeneous mix of cells at different stages of cancer development, and if targeted drugs can only be effective at particular stages, then using a single targeted drug might only eliminate a fraction of cancer cells. Thus it will probably take multiple targeted drugs to completely eliminate most cancer types. Incoming results from the recently established Cancer Genome Atlas Project indicate that a typical solid tumor carries as many as 30–80 somatic mutations [72], suggesting difficulties faced trying to choose the right combination of targets in most cancer types. In the future, however, genome sequencing of cancer cells from individuals would enable us to identify all the somatic mutations for each individual cancer. Protein profiles of these same tumor cells could also be built. Based on the signaling pathways determined from these data, the treatment for each individual could be customized. This would be the height of personalized medicine, but even in the future, this could be an enormously expensive option.

Despite the advances made in personalized medicine, we believe the ideal cancer therapy should still be the therapy that is applicable to all sorts of cancer types at many stages of development, including highly metastasized late stages of cancer. We believe the ideal cancer therapy may be possible by combining deoxyglucose with drugs targeting mitochondria for direct mechanical activation of mitochondrial outermembrane pores (MOMP). Once MOMP is activated, cytochrome c is released into cytosol, activating apoptosomes, the death executioner. Thus by activating steps very close to the final step in apoptosis, we are lowering the risks of cancerous cells developing therapy-resistant mutations.

There are two unique features of deoxyglucose that makes it an ideal chemotherapeutic partner. The first is the almost exclusive uptake of deoxyglucose by cancer cells (see the introduction). The second feature is that it primes cells for mitochondria-dependent apoptosis. Bak is an important component of mitochondrial outermembrane pores (MOMP). It is normally sequestered in complexes with Mcl-1 and Bcl-xL. We have shown that in cells treated with deoxyglucose, Mcl-1 dissociates from Bak, leaving only Bcl-xL to sequester Bak from forming MOMPs. Antagonists of the Bcl-2 family, such as ABT-263 and ABT-737, likely dissociate Bcl-xL from Bak, inducing apoptosis at a submicromolar concentration.

Deoxyglucose-ABT-263 combination therapy was shown to be effective in eliminating cancer cells representing many different cancer types and covering different stages of development. Using human tumor cells xenografted onto nude mice, we showed that the combination therapy induced tumor regression in checkpoint defective (point mutation in p53), chemo-resistant, PTEN deleted, late stage cancer cells. In mice, the combination therapy specifically targeted tumor cells and eliminated them safely and more efficiently than 2DG or ABT alone. Even though 2DG-ABT combination therapy is still at an early stage of development as cancer therapy, it is meeting all the criteria for an all purpose-multifaceted cancer therapy. Thus we are hopeful that we may have found a simple solution to a very complex problem of treating cancer.

## Materials and Methods

### Reagents

2-Deoxy-D-glucose was purchased from Sigma (D3179). 

Ethyl 3-Bromopyruvate was purchased from Lancaster Chemicals (Cat. L00582). 

z-VAD-fmk (benzoyl-Valinyl-Alaninyl-Aspartyl-fluoromethyl-ketone) was purchased from BD Bioscience. 

To stimulate death-receptor pathway in HT1080 cells, we used anti-Fas mouse antibody from MBL, clone CH-11 (CD95), 0.5 mg/ml used at 3 and 0.3 µL/ml. 

ABT-737 was purchased from Selleck Chemicals or provided by Abbott Laboratories, Inc. 

ABT-263 was purchased from Chimietech. 

Staurosporine, Thapsigargin, and Tunicamycin, were purchased from EMD Bioscience. 

PI3K inhibitor, LY294002, was purchased from Merk Chemicals.

### Cell lines

Prof. Koji Yamada, Kyushu University, generously provided **MCF7, HepG2 and PANC-1**. **Bid KO MEF** cells were a kind gift from Prof. Atan Gross, The Weizmann Institute of Science. **NCI H82** cells were kind gifts from Prof. Jun Yokota, National Cancer Research Center, Tokyo. **RS(4;11)** cells was a kind gift from Prof. Hidemistu Kurosawa, Dept. of Pediatrics, Dokkyo Medical University, Mibu, Tochigi, Japan. **Prostate cancer cell line PPC-1/PC-3** (Cytogenet Cell Genet 1993;62:183-4), and all other cells were from Sanford-Burnham Medical Research Institute.

All the experiments in the article were performed using either ABT-737 or ABT-263, and then repeated using the other agent, except for the mouse xenograft work and the EM work for which only ABT-263 were used.

### Treatment of cancer cells with 2DG and ABT-263/737

We tested several conditions such as the length of pre-incubation and the concentration of deoxyglucose in the media (Fig. S2 and data not shown), and developed the following protocol. To cells cultured in appropriate medium, add 2DG at a molar ratio 0.5∶1.0 2DG to glucose, and incubate the cells for three hours before ABT addition. In DMEM that contains 2 g/L of glucose, we typically used 10 mM 2DG, while in high glucose medium, we used 20 mM 2DG. We regularly used 1 µM ABT-263/737 for the 2DG-ABT combination for cancer cells and 3 µM for MEFs. For a single-agent application, we used 10 µM ABT for cancer cells and 30 µM ABT for MEFs.

### Western blotting and immunoprecipitation

We usually run either 12.5% or 4–20% gradient gels for Western Blots, except for detecting loss of long isoforms for Opa1 (8% SDS-PAGE gels), and separating Bak protein from IgG Light Chain (15% SDS-PAGE gels). The following antibodies were used for Western Blots: **anti-Opa1** mouse mAb from Transduction Lab (Cat. 612606), **anti-Cyt C** mouse mAb from BD Bioscience (Cat 556433), **anti-Bid** mouse mAb from Santa Cruz (5C9) 0.1 mg/ml, **anti-Bid** Rabbit Polyclonal Ab from Thermo Scientific (Cat PA1-84831), **anti-Bak NT** Rabbit polyclonal Ab from Millipore, **anti-Bax** and **anti-Mcl-1** Rabbit Polyclonal Abs were previously described [Krajewski, et al 1994 *Am. S. Pathol* 145–515; Krajewski S et al 1995 *Cancer Res* 55: 4471], **anti-Grp78** Rabbit Polyclonal Ab from ABR (Cat. PA1-014), **anti-PKM2** antibody gifted from the Lewis Cantley laboratory. To immunoprecipitate of Bak and Mcl-1 proteins from PPC-1 cells, we used mitochondria-enriched fraction [20], re-suspended in buffer containing 1% Triton X-100 as described [20]. To immunoprecipitate Bak and Mcl-1 proteins, antibodies against them (anti-Bak NT Rabbit polyclonal Ab from Millipore, and Anti-Mcl1 Rabbit Polyclonal Ab from Krajewski) were conjugated to Protein G-Sepharose, mixed with lysates, and incubated overnight on a rotating platform. To separate Bak from IgG Light Chain, we run immunoprecipitates in 15% SDS-PAGE. For detection of Mcl-1, we run precipitates in 12.5% SDS-PAGE.

### Immunofluorescence microscopy

Cells were treated as described [31]. Briefly, cells were fixed in 3.7% formaldehyde for 20 min, washed in PBS, and permeabilized with 0.5% Triton X-100. Nonspecific binding was blocked by incubating cells in 0.2% gelatin/PBS for 30 min. Cytochrome c staining was performed using a primary antibody against cytochrome c (clone 6H2.B4, Cat 556432, BD Science) and a fluorescent secondary antibody conjugate, goat anti-mouse IgG, AlexaFluor 568 (Molecular Probes). Images were captured by a MRC 1024 SP BioRad Laser Point Scanning Confocal Microscope. For Bax translocation studies, we used anti-Bax Rabbit Polyclonal Ab [Krajewski, et al 1994 *Am. S. Pathol* 145–515; Krajewski S et al 1995 *Cancer Res* 55: 4471].

### Electron microscopy

Cells were prepared and the electron microscopy was performed following the procedure described in “Elevated hydrostatic pressure triggers release of OPA1 and cytochrome C, and induces apoptotic cell death in differentiated RGC-5 cells”, [Ju WK, Kim KY, Lindsey JD, Angert M, Patel A, Scott RT, Liu Q, Crowston JG, Ellisman MH, Perkins GA, Weinreb RN. Mol Vis. 2009;15:120-34.]

### Bid siRNA

From Qiagen Cat. No SI02661911 (5′-AGACAAUGUUAAACUUAUATT-3′ & 5′-UAUAAGUUUAACAUUGUCUTT-3′) HeLa and PPC-1 cells were transfected with siRNA above and using Lipofectamine 2000 (invitrogen) using the manufacture's protocol. We used mock transfection for controls. For both cells, transfections were done twice, 24 hours apart. Cells were used a day later for western blots and apoptosis assays.

### Apoptosis assay

RS(4;11) cells were stained with Annexin V-FITC (Invitrogen PHN1008) and analyzed by FACSort (BD). HeLa cells were stained with Annexin V-APC (Invitrogen A35110) and analyzed by BDFACSCanto according to the manufacture's instruction. Yoav Alterman and Shinichi Kitada at Sanford-Burnham Medical Research Institute performed FACS analysis.

### ATP measurement

ATPlite solution (10 µL for 200 µL cell culture) was added, and a luminomitor (Monolight 3096; BD Biosciences) was used to measure luminescence.

### Caspase assay

Cells grown to 80% confluency in 6 well dishes (diameter 34.9 mm) were washed in PBS and then incubated in appropriate media (e.g., DMEM with 12 mM glucose for HeLa cells). 5–10 mM 2DG was added to the indicated samples, and 3 hours later, 1 µM ABT-737 was added to the indicated samples. 3 hours later, cells were (1) washed once in PBS, (2) incubated in 0.05% trypsin, 0.2 g/L EDTA in Hank's Balanced Salt, (3) diluted in 10× volume DMEM containing 10% serum and trypsin inhibitor, (4) cells were span-down by centrifugation), (5) washed once with PBS, (6) pellets were re-suspended in 100 µL lysis buffer [20 mM Hepes, pH 7.4, 0.5% Triton X-100, 0.5 mM EDTA, and 150 mM NaCl] , (7) incubated on ice for 10 minutes, and then centrifuged at 10,000 g to remove the insoluble fraction. 10 µL of supernatant was diluted in 100 µL buffer [50 mmol/L HEPES (pH 7.4), 100 mmol/L NaCl, 0.1% CHAPS, 10 mmol/L DTT, 1 mmol/L EDTA, and 10% glycerol], containing 100 µmol/L Ac-DEVD-AFC and caspase activities were measured by a spectrofluorimeter at 37°C taking measurement for 20 min. Increases in relative fluorescence units [RFU] per minute were determined from the graph.

### Cell fractionation

Cell were first washed in PBS twice, then re-suspended in buffer containing 10 mM Hepes (pH 7.4) 10 mM KCl, 1 mM EDTA, 1 mM EGTA and 250 mM trehalose, at one million cells per 0.5 ml. They were incubated on ice for 30 min. Cells were dounce or needle homogenized until a small sample of cells showed roughly 40% trypan blue positive. Cells were centrifuged at 10,000 g for 5 minutes for three times, each time collecting the supernatant. The supernatant was almost exclusively cytosolic fractions.

### Statistical analysis

Statistical analysis was performed at Graphpad, http://www.graphpad.com/quickcalcs/ttest1.cfm?Format=C.

### Mouse xenograft

Mice were maintained under specific pathogen-free conditions in accordance with guidelines by the Association for Assessment and Accreditation of Laboratory Animal Care International.

CCHMC is credited through the Animal Welfare Assurance (#A3108-01). All animal experiments were carried out in strict accordance with animal protocol #8C03021 that was approved on 3/10/2008 by the institutional IACUC that operates according with the guidelines by the Association for Assessment and Accreditation of Laboratory Animal Care International and the recommendations in the Care and Use of Laboratory Animals of the National Institute of Health.

Eight-week-old male Athymic Nude mice (Hsd:Athymic Nude-*Fox1^nu^*, Harlan, Indiana, USA) were engrafted with 5×10^6^ PPC-1 cells in 0.1 ml 50% matrigel (BD Bioscience) s.c. in the upper left flank. Nine days later tumor-bearing mice were randomly divided over all treatment groups. ABT-263 was formulated in 10% ethanol, 90% Cremophor (Sigma) and glucose/2-Deoxyglucose in PBS. Mice received either Cremophor only (0.2 ml i.p.), ABT-263 (2 mg/0.2 ml i.p.), 2-deoxyglucose (10 mg/0.2 ml i.p.), or a mix of glucose/deoxyglucose (10 mg each in a total volume of 0.2 ml i.p.). For combination treatments, ABT-263 was given 3 hr after the other agents. Tumor size was measured two to three times weekly by electronic calipers (volume = (length×width^2^)/2). All studies used 5–10 mice per group. Statistical comparisons of tumor growth rate and survival used the Wilcoxon rank sum test and the Mantle-Cox log-rank test respectively. Mice were maintained under specific pathogen-free conditions in accordance with guidelines by the Association for Assessment and Accreditation of Laboratory Animal Care International.

## Supporting Information

Figure S1 **Effects of 2DG on ABT-263 induced apoptosis.** **a,** RS(4;11) cells pre-treated with 20 mM fructose or 10 mM 2-deoxy-D-glucose in regular RPMI medium containing 12 mM glucose, for 1 hour before the addition of ABT-263 at indicated concentrations. 24 hours later, cells were analyzed by trypan-blue dye exclusion assay. **b,** HeLa cells were pre-treated for 1 hour either with or without 10 mM deoxyglucose in DMEM containing 12 mM glucose, before adding indicated amounts of ABT-263, or 1 µM STS. 24 hours later, cells were assayed by trypan-blue dye exclusion assay. % of blue dead cells were tallied against 100% input.
(TIFF)

Figure S2 **Optimizing conditions for 2DG-ABT induced apoptosis. a, Comparable sensitization of tumor cells to ABT-737 and ABT-263 by 2DG.** HeLa cells were pre-treated with 10 mM 2DG for 3 hours, then treated with either ABT-737 or ABT-263 for 3 hours at the indicated concentrations (µM). Caspase activities were compared. Data represent results of triplicate samples (mean ± std dev; n = 3). **b, Conditions for 2DG treatment.** HeLa cells grown to 80% confluency in 6 well dishes (diameter 34.9 mm) were washed in PBS and incubated in 10% serum in DMEM without glucose or with 2 g/L of glucose (12 mM glucose). Immediately, 10 mM 2DG was added to the indicated samples, and 3 hours later, 1 µM ABT-737 was added to the indicated samples. 3 hours later, cells were harvested and caspase activities were measured (relative fluorescence units [RFU] per min per×mg of lysates, see Methods). **c,** HeLa cells were pre-incubated with 10 mM 2DG for indicated lengths of time before 1 µM ABT-263 or 1 µM STS was added. Caspase activities were measured three hours later. All data represent mean ± std dev (n = 3). **d,** HeLa cells were pre-treated with 10 mM 2DG for indicated length of time before the addition of 1 µM ABT-737. 24 hours later, dead cells were counted by Trypan Bleu Inclusion Assay, and % of dead cells were graphed.
(TIFF)

Figure S3 **Cytochrome c release is by 2DG-ABT and is blocked by z-VAD.** HeLa cells were treated with or without 2DG for three hours, followed by 1 µM ABT-737+/−10 µM z-VAD for 3 hours. Cells were fixed and nuclei were stained by propidium iodine (red), while cytochrome c was stained with anti-cytochrome c antibody followed by FITC-conjugated secondary antibody (green).
(TIFF)

Figure S4 **Deciphering how 2DG is priming cancer cells for ABT-induced apoptosis. a, Bax translocates to mitochondria after 16 hour-incubation with 10 mM 2DG.** HeLa cells were treated with 10 mM 2DG in the presence of 12 mM glucose for 0–16 hours. Cells were fixed and examined at 0, 4, 8 and 16 hour point, and examined for translocated Bax. % of cells with mitochondrial Bax was graphed (Left). Represetative images of diffused cytosolic Bax (right upper panel) and mitochondria-localized Bax (right lower Panel) were shown. **b & c, analysis of glycolysis inhibitors and role of ER stress. b,** HeLa cells were incubated with 10 nM Thapsigargin or 5 µg/ml Tunicamycin in media containing ^35^S-methionine, for 0–8 hours. Total lysates were fractionated by 4–16% SDS-PAGE, transferred to membrane and exposed to x-ray film. Induction of ER stress marker, Grp74, is seen at 4–8 hours. The identity of the band was also confirmed by immunoblotting using a Grp74-specific antibody. We did not detect any other changes in these samples. **c, Thapsigirgin did not prime HeLa cells for ABT-induced apoptosis.** HeLa cells were either treated with 10 nM Thapsigargin for 4 hours or left untreated before addition of 0–3 µM ABT-737 or 1 µM STS. Caspase activities were measured 3 hrs later (mean ± std dev; n = 3). **d, Effects of 2DG & ABT on Mcl-1 on various cell lines.** Cancer cells, PPC1, MCF-7, HepG2 and RS(4;11) were treated with 10 mM 2DG for 0–6 hours, 1 µM ABT-263 for 3 hours (ABT only), or pre-treated with 2DG for 3 hours followed by the addition of 1 µM ABT-263 for 3 hours (Both). Samples were analyzed by Western blot for Mcl-1 and a-tubulin.
(TIFF)

Figure S5 **The factors that affect 2DG-ABT induced apoptosis. a, Effects of 2DG, ABT-263 and combination treatments on MEFs.** Wt and Bax/Bid single KO MEFs and Bax/Bak DKO MEFs were treated with or without 2DG for 3 hours, then 3 µM ABT-263 was added in indicated cells, and 24 hours later, viable cells were counted by trypan blue dye exclusion methods. **b, PI3K inhibitor enhanced 2DG-ABT induced apoptosis.** HeLa cells were treated with or without 10 µM LY294002 (PI3K inhibitor) for 5 minutes. Then cells were subjected to the standard 2DG/ABT-263 treatment. Cells were harvested 3 hours after ABT addition and caspase activity was assayed.
(TIFF)

Figure S6 **Additional mouse xenograft data.** The experiment presented in Fig. 7 was repeated with a larger number of mice for each group. Otherwise, all the materials and conditions were the same. **a,** Tumor volume was measured using external calipers for each mouse. The rectangle indicates the period during which drug treatment was administered. **b,** The percentage of surviving mice is indicated.
(TIFF)

Information S1 (DOC)
