# Supplementary figures and images for: Efficient Elimination of Cancer Cells by Deoxyglucose-ABT-263/737 Combination Therapy
Source: PLoS One. 2011 Sep 19;6(9):e24102. doi: 10.1371/journal.pone.0024102 (PMC3176271; doi:10.1371/journal.pone.0024102)

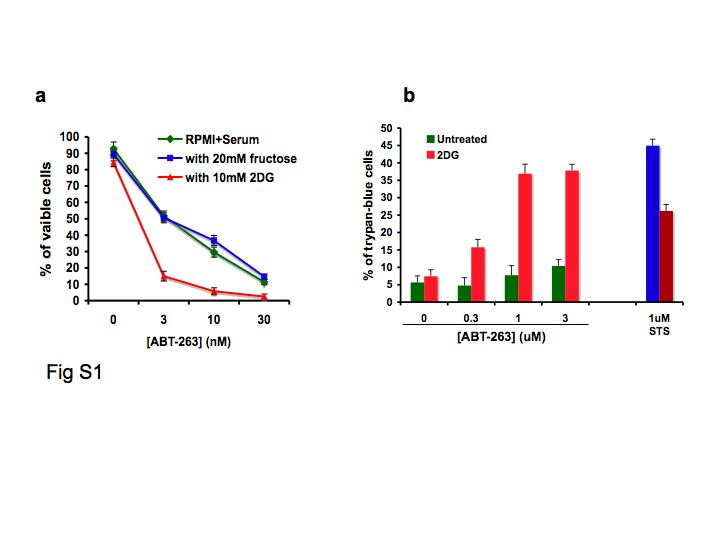

Supplement: Figure S1 — Effects of 2DG on ABT-263 induced apoptosis. a, RS(4;11) cells pre-treated with 20 mM fructose or 10 mM 2-deoxy-D-glucose in regular RPMI medium containing 12 mM glucose, for 1 hour before the addition of ABT-263 at indicated concentrations. 24 hours later, cells were analyzed by trypan-blue dye exclusion assay. b, HeLa cells were pre-treated for 1 hour either with or without 10 mM deoxyglucose in DMEM containing 12 mM glucose, before adding indicated amounts of ABT-263, or 1 µM STS. 24 hours later, cells were assayed by trypan-blue dye exclusion assay. % of blue dead cells were tallied against 100% input. (TIFF) [file pone.0024102.s001.tiff]

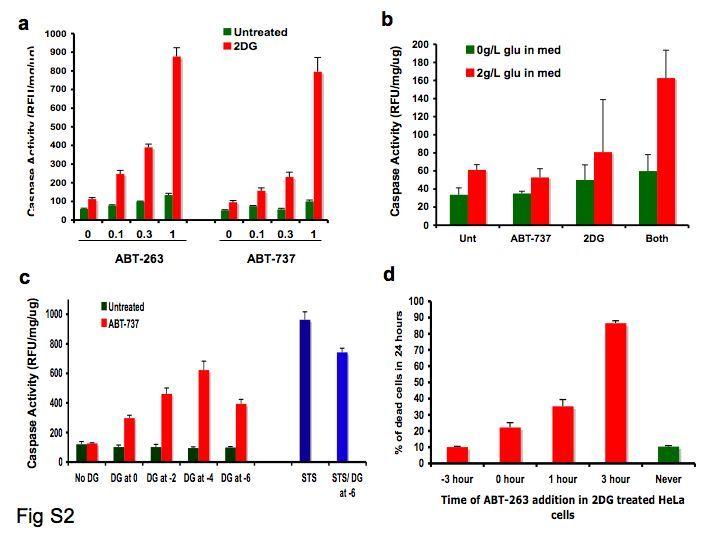

Supplement: Figure S2 — Optimizing conditions for 2DG-ABT induced apoptosis. a, Comparable sensitization of tumor cells to ABT-737 and ABT-263 by 2DG. HeLa cells were pre-treated with 10 mM 2DG for 3 hours, then treated with either ABT-737 or ABT-263 for 3 hours at the indicated concentrations (µM). Caspase activities were compared. Data represent results of triplicate samples (mean ± std dev; n = 3). b, Conditions for 2DG treatment. HeLa cells grown to 80% confluency in 6 well dishes (diameter 34.9 mm) were washed in PBS and incubated in 10% serum in DMEM without glucose or with 2 g/L of glucose (12 mM glucose). Immediately, 10 mM 2DG was added to the indicated samples, and 3 hours later, 1 µM ABT-737 was added to the indicated samples. 3 hours later, cells were harvested and caspase activities were measured (relative fluorescence units [RFU] per min per×mg of lysates, see Methods). c, HeLa cells were pre-incubated with 10 mM 2DG for indicated lengths of time before 1 µM ABT-263 or 1 µM STS was added. Caspase activities were measured three hours later. All data represent mean ± std dev (n = 3). d, HeLa cells were pre-treated with 10 mM 2DG for indicated length of time before the addition of 1 µM ABT-737. 24 hours later, dead cells were counted by Trypan Bleu Inclusion Assay, and % of dead cells were graphed. (TIFF) [file pone.0024102.s002.tiff]

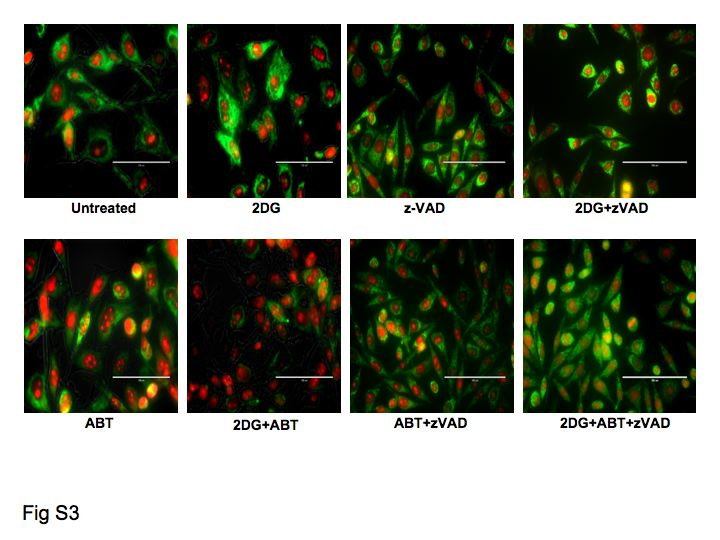

Supplement: Figure S3 — Cytochrome c release is by 2DG-ABT and is blocked by z-VAD. HeLa cells were treated with or without 2DG for three hours, followed by 1 µM ABT-737+/−10 µM z-VAD for 3 hours. Cells were fixed and nuclei were stained by propidium iodine (red), while cytochrome c was stained with anti-cytochrome c antibody followed by FITC-conjugated secondary antibody (green). (TIFF) [file pone.0024102.s003.tiff]

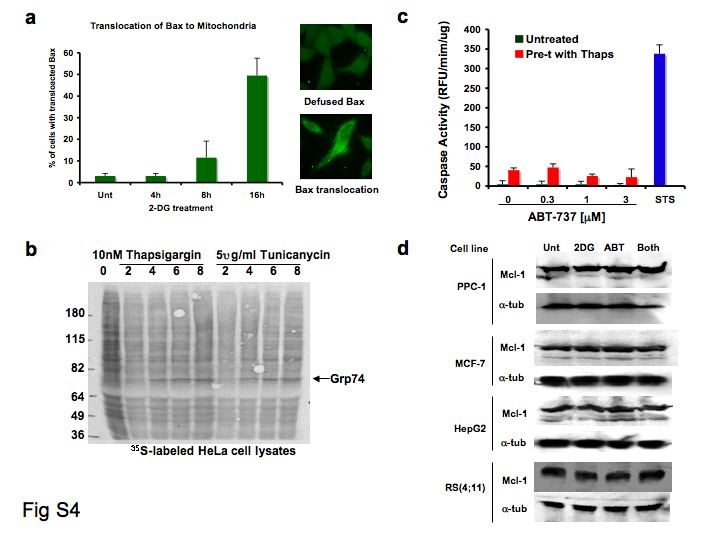

Supplement: Figure S4 — Deciphering how 2DG is priming cancer cells for ABT-induced apoptosis. a, Bax translocates to mitochondria after 16 hour-incubation with 10 mM 2DG. HeLa cells were treated with 10 mM 2DG in the presence of 12 mM glucose for 0–16 hours. Cells were fixed and examined at 0, 4, 8 and 16 hour point, and examined for translocated Bax. % of cells with mitochondrial Bax was graphed (Left). Represetative images of diffused cytosolic Bax (right upper panel) and mitochondria-localized Bax (right lower Panel) were shown. b & c, analysis of glycolysis inhibitors and role of ER stress. b, HeLa cells were incubated with 10 nM Thapsigargin or 5 µg/ml Tunicamycin in media containing 35S-methionine, for 0–8 hours. Total lysates were fractionated by 4–16% SDS-PAGE, transferred to membrane and exposed to x-ray film. Induction of ER stress marker, Grp74, is seen at 4–8 hours. The identity of the band was also confirmed by immunoblotting using a Grp74-specific antibody. We did not detect any other changes in these samples. c, Thapsigirgin did not prime HeLa cells for ABT-induced apoptosis. HeLa cells were either treated with 10 nM Thapsigargin for 4 hours or left untreated before addition of 0–3 µM ABT-737 or 1 µM STS. Caspase activities were measured 3 hrs later (mean ± std dev; n = 3). d, Effects of 2DG & ABT on Mcl-1 on various cell lines. Cancer cells, PPC1, MCF-7, HepG2 and RS(4;11) were treated with 10 mM 2DG for 0–6 hours, 1 µM ABT-263 for 3 hours (ABT only), or pre-treated with 2DG for 3 hours followed by the addition of 1 µM ABT-263 for 3 hours (Both). Samples were analyzed by Western blot for Mcl-1 and a-tubulin. (TIFF) [file pone.0024102.s004.tiff]

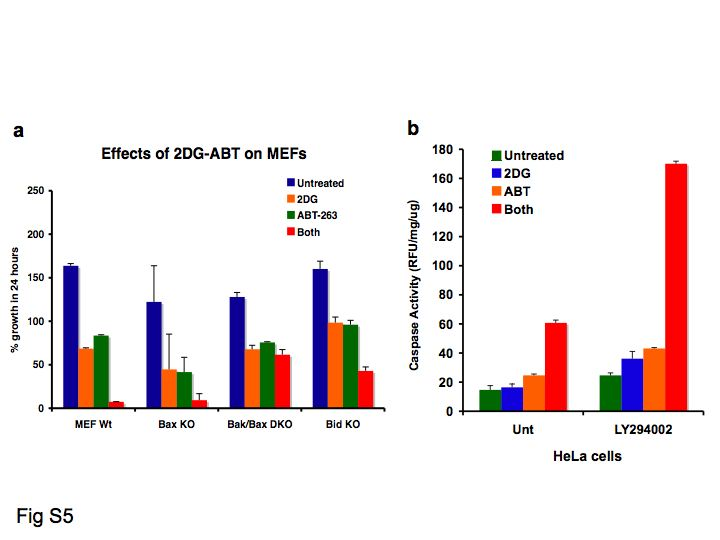

Supplement: Figure S5 — The factors that affect 2DG-ABT induced apoptosis. a, Effects of 2DG, ABT-263 and combination treatments on MEFs. Wt and Bax/Bid single KO MEFs and Bax/Bak DKO MEFs were treated with or without 2DG for 3 hours, then 3 µM ABT-263 was added in indicated cells, and 24 hours later, viable cells were counted by trypan blue dye exclusion methods. b, PI3K inhibitor enhanced 2DG-ABT induced apoptosis. HeLa cells were treated with or without 10 µM LY294002 (PI3K inhibitor) for 5 minutes. Then cells were subjected to the standard 2DG/ABT-263 treatment. Cells were harvested 3 hours after ABT addition and caspase activity was assayed. (TIFF) [file pone.0024102.s005.tiff]

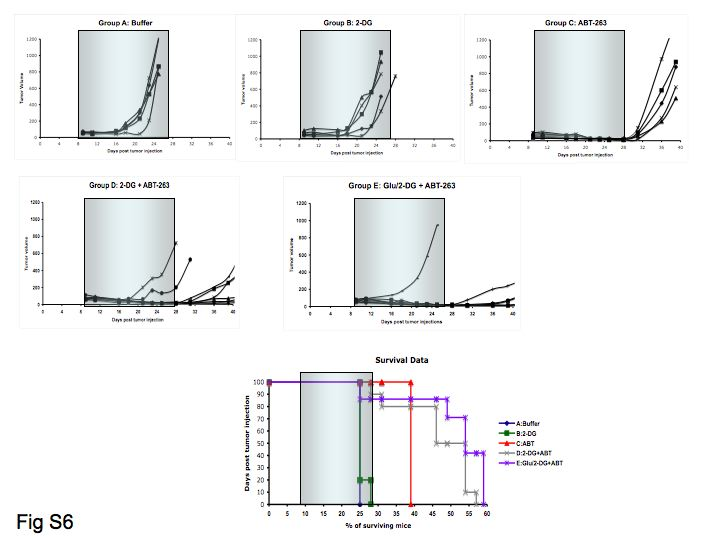

Supplement: Figure S6 — Additional mouse xenograft data. The experiment presented in Fig. 7 was repeated with a larger number of mice for each group. Otherwise, all the materials and conditions were the same. a, Tumor volume was measured using external calipers for each mouse. The rectangle indicates the period during which drug treatment was administered. b, The percentage of surviving mice is indicated. (TIFF) [file pone.0024102.s006.tiff]
