# Supplementary material for: Efficient Elimination of Cancer Cells by Deoxyglucose-ABT-263/737 Combination Therapy
Source: PLoS One. 2011 Sep 19;6(9):e24102. doi: 10.1371/journal.pone.0024102 (PMC3176271; doi:10.1371/journal.pone.0024102)
Supplement: Information S1 — (DOC) [file pone.0024102.s007.doc]

**Supplemental Information S1.**

(1) Certo M, Del Gaizo Moore V, Nishino M, et al. Mitochondria primed by death signals determine cellular addiction to antiapoptotic BCL-2 family members. Cancer Cell 2006;9:351-65.

(2) Chen L, Willis SN, Wei A, et al. Differential targeting of prosurvival Bcl-2 proteins by their BH3-only ligands allows complementary apoptotic function. Mol Cell 2005;17:393-403.

(3) Chen S, Dai Y, Harada H, Dent P, Grant S. Mcl-1 down-regulation potentiates ABT-737 lethality by cooperatively inducing Bak activation and Bax translocation. Cancer Res 2007;67:782-91.

(4) Dai Y, Grant S. Targeting multiple arms of the apoptotic regulatory machinery. Cancer Res 2007;67:2908-11.

(5) Hauck P, Chao BH, Litz J, Krystal GW. Alterations in the Noxa/Mcl-1 axis determine sensitivity of small cell lung cancer to the BH3 mimetic ABT-737. Mol Cancer Ther 2009;8:883-92.

(6) Keuling AM, Felton KE, Parker AA, Akbari M, Andrew SE, Tron VA. RNA silencing of Mcl-1 enhances ABT-737-mediated apoptosis in melanoma: role for a caspase-8-dependent pathway. PLoS One 2009;4:e6651.

(7) Lin X, Morgan-Lappe S, Huang X, et al. 'Seed' analysis of off-target siRNAs reveals an essential role of Mcl-1 in resistance to the small-molecule Bcl-2/Bcl-XL inhibitor ABT-737. Oncogene 2007;26:3972-9.

(8) van Delft MF, Wei AH, Mason KD, et al. The BH3 mimetic ABT-737 targets selective Bcl-2 proteins and efficiently induces apoptosis via Bak/Bax if Mcl-1 is neutralized. Cancer Cell 2006;10:389-99.

(9) Konopleva M, Contractor R, Tsao T, et al. Mechanisms of apoptosis sensitivity and resistance to the BH3 mimetic ABT-737 in acute myeloid leukemia. Cancer Cell 2006;10:375-88.

(10) Vogler M, Dinsdale D, Sun XM, et al. A novel paradigm for rapid ABT-737-induced apoptosis involving outer mitochondrial membrane rupture in primary leukemia and lymphoma cells. Cell Death Differ 2008;15:820-30.
